# Effect of gastric distension with concurrent small intestinal saline or glucose infusion on incretin hormone secretion in healthy individuals: A randomized, controlled, crossover study

**DOI:** 10.1111/dom.15042

**Published:** 2023-03-17

**Authors:** Ryan J. Jalleh, Laurence G. Trahair, Tongzhi Wu, Scott Standfield, Christine Feinle‐Bisset, Christopher K. Rayner, Michael Horowitz, Karen L. Jones

**Affiliations:** ^1^ Adelaide Medical School The University of Adelaide Adelaide Australia; ^2^ Centre of Research Excellence in Translating Nutritional Science to Good Health Adelaide Australia; ^3^ Endocrine and Metabolic Unit Royal Adelaide Hospital Adelaide Australia; ^4^ Diabetes and Endocrine Services Northern Adelaide Local Health Network Adelaide Australia; ^5^ Department of Gastroenterology and Hepatology Royal Adelaide Hospital Adelaide Australia

**Keywords:** barostat, gastric distension, glucagon‐like peptide‐1, glucose‐dependent insulinotropic polypeptide, incretin, intragastric balloon

## Abstract

**Aim:**

To evaluate the effect of gastric distension, induced using a gastric ‘barostat’, on the secretion of glucose‐dependent insulinotropic polypeptide (GIP) and glucagon‐like peptide‐1 (GLP‐1) in the presence and absence of small intestinal nutrients in healthy individuals.

**Materials and Methods:**

Eight healthy participants (two females, six males, mean age 69.3 ± 1.2 years, body mass index 23.5 ± 0.8 kg/m^2^) were each studied on four occasions when they received an intraduodenal infusion of either (i) 0.9% saline or (ii) glucose delivered at a rate of 3 kcal/min both with, and without, an intragastric balloon with the pressure set to 8 mmHg above the intragastric minimum distending pressure.

**Results:**

Following intraduodenal saline or glucose infusion, there was no difference in plasma GLP‐1 with or without gastric distension (*P* = 1.00 for both saline and glucose infusions). There was also no difference in plasma GIP with or without gastric distension (*P* = 1.00 for saline infusion and *P* = .99 for glucose infusion).

**Conclusions:**

Gastric distension, either alone or during small intestinal glucose exposure, does not stimulate incretin hormone secretion significantly in healthy humans.

## 1 INTRODUCTION

The use of intragastric balloons as a therapy for obesity was initially explored in the mid‐1980s, but following the negative outcome of a sham‐controlled trial and evidence of substantial complications, it was not evaluated further.^1^ Decades later, interest in this therapy for obesity has re‐emerged.^2^, ^3^ Newer balloon designs are safer,^2^, ^3^ and may be ‘procedureless’ (i.e. swallowable)^2^ or adjustable,^3^ while the reversibility of this therapy is an advantage over metabolic surgery. Furthermore, the use of an intragastric balloon may also be an effective ‘bridging therapy’ for people with obesity when urgent weight reduction is indicated to reduce operative risk.^4^ However, the fundamental question of how gastric distension induces weight loss remains unknown. Increased secretion of incretin hormones, in particular glucagon‐like peptide‐1 (GLP‐1), is believed to be a key mediator for weight loss and remission of type 2 diabetes following metabolic surgery, and represents a potential mechanism for the effectiveness of intragastric balloons.^5^, ^6^ The concept that non‐nutrient gastric distension could stimulate GLP‐1 has recently been shown in rodent studies. Natochin et al.^7^ reported that intragastric administration of water and sodium chloride stimulated plasma GLP‐1 markedly within 15 minutes, as did gastric balloon distension in anaesthetised rats. In unanesthetized mice, Ohbayashi et al.^8^ reported that gastric distension with a pectin‐containing carbonated solution resulted in a load‐dependent, sustained, substantial stimulation of portal concentrations of GLP‐1. It has been suggested that effects on vagal function may underlie the stimulation of GLP‐1. There is no information about the effect of gastric distension on the secretion of the other incretin hormone, glucose‐dependent insulinotropic polypeptide (GIP), in animals.

In contrast to the robust evidence of a major effect of gastric distension on GLP‐1 secretion in rodents, it is not known whether gastric distension affects either GLP‐1 and/or GIP secretion in humans. That Steinert et al.^9^ observed that intragastric infusion of glucose stimulated plasma GLP‐1 more than an equivalent amount of intraduodenally infused glucose in healthy individuals, suggests that gastric signalling may potentiate nutrient‐induced GLP‐1 secretion.

As incretin hormones, GIP and GLP‐1 have a major role in postprandial glucose homeostasis.^10^ GIP is secreted predominantly by K‐cells in the proximal small intestine and GLP‐1 by L‐cells in the distal small intestine and colon.^11^ GIP and GLP‐1 are released in response to all three macronutrients^12^ following nutrient entry into the small intestine and subsequent digestion, and the stimulation of GLP‐1 in turn inhibits subsequent gastric emptying,^13^ suppresses appetite and reduces energy intake.^14^ In contrast to GLP‐1, GIP neither slows gastric emptying^15^ nor appears to reduce energy intake in humans^14^; however, a co‐agonist of both GLP‐1 and GIP, tirzepatide, has recently been shown to promote greater weight loss than GLP‐1 mono‐agonists.^16^ Accordingly, stimulation of incretin hormones, perhaps particularly GLP‐1, represents an important mechanism for inducing weight loss. Nutrient preloads of glucose, protein or fat stimulate the secretion of endogenous incretins, but at the cost of additional energy intake.^17^, ^18^, ^19^ A non‐nutritive strategy to enhance incretin secretion would be highly desirable.

The purpose of this study was to evaluate the effects of gastric distension, using a gastric barostat, on the secretion of GIP and GLP‐1 in the presence and absence of small intestinal nutrients in healthy individuals. While gastric distension may potentially have a different effect on incretin hormones in individuals with obesity, we considered that an understanding of normal physiology represented an appropriate initial step.

## 2 MATERIALS AND METHODS

### 2.1 Participants

Eight healthy older participants (two females and six males, mean age 69.3 ± 1.2 years, body mass index [BMI] 23.5 ± 0.8 kg/m^2^) were recruited. All participants were non‐smokers and none had a history of cardiac, respiratory, gastrointestinal (or prior gastrointestinal surgery), hepatic or renal disease. None had a history of diabetes, epilepsy or an intake of more than 20 g of alcohol per day. No participant took medication known to influence gastrointestinal function. This is a secondary analysis and data from this cohort relating to the effects of gastric distension and small intestinal nutrients on blood pressure and superior mesenteric artery blood flow have been reported.^20^

### 2.2 Protocol

Participants were studied on four occasions in a randomized order following an overnight fast (10 hours for solids, 8 hours for liquids), separated by at least 3 days. Upon arrival at the laboratory at the Royal Adelaide Hospital, a silicone‐rubber, multilumen nasoduodenal catheter (Dentsleeve International, Mui Scientific, Mississauga, Canada) was inserted into the stomach via an anaesthetized nostril and allowed to pass into the duodenum by peristalsis. The catheter comprised an infusion channel (internal diameter ~ 1 mm) located ~ 10 cm distal to the pylorus with two other channels, located 2.5 cm either side of the pylorus, perfused continuously with 0.9% saline, allowing continuous measurement of the transmucosal potential difference from the antral (−40 mV) and duodenal (0 mV) channels to ensure correct positioning of the catheter.^21^, ^22^ An intravenous cannula was positioned in the antecubital fossa of one arm for blood sampling.

On two of the four study days, the participant also swallowed a single‐lumen polyvinyl orogastric catheter (external diameter ~ 4 mm) (Tygon tubing, Saint Gobain Performance Plastics, Akron, OH) equipped with a thin, flaccid polyethylene bag (capacity 1200 ml) that was tightly wrapped around the distal end. The proximal end of the catheter was connected to a gastric barostat (Distender Series II, G&J Electronics, Willowdale, ON, Canada). The barostat bag was inflated by 1 mmHg every 5 minutes to determine the intragastric minimum distending pressure (MDP), which represents the minimum pressure required to overcome the intra‐abdominal pressure and is defined as the pressure to achieve a volume of more than 30 ml in the bag.^20^, ^23^ The stomach was then distended using a stepwise increase in intragastric pressure by 2‐mmHg increments every 3 minutes, in four steps, to achieve a distension of 8 mmHg above MDP.^24^ Between *t* = 0‐60 minutes, subjects received an intraduodenal infusion of either (a) 0.9% saline, (b) 25% glucose at 3 kcal/min, (c) 0.9% saline with intrabag pressure set to 8 mmHg above MDP or (d) glucose at 3 kcal/min with intrabag pressure set to 8 mmHg above MDP in a computer‐generated randomized order. At this rate of glucose infusion, there would be substantial stimulation of incretin hormones.^25^ Participants and investigators were blinded to whether saline or glucose was infused. At *t* = 60 minutes the barostat bag was deflated and between *t* = 60‐120 minutes 0.9% saline was infused intraduodenally.^20^, ^22^ Intraduodenal infusions were performed at a rate of 3 ml/min.

### 2.3 Data collection

Venous blood samples (~ 18 ml) were obtained at 15‐minute intervals between *t* = 0‐120 minutes. Blood samples were centrifuged at 3200 rpm for 15 minutes and plasma or serum was separated and stored at −70°C for subsequent analysis.

### 2.4 Ethics

The protocol was approved by the Research Ethics Committee of the Royal Adelaide Hospital, and was performed in accordance with the Declaration of Helsinki. Each subject provided written, informed consent.

### 2.5 Laboratory methods

#### 2.5.1 Blood glucose

Blood glucose concentrations (mmol/L) were determined immediately using a portable blood glucose meter (Precision QID System, Abbott Laboratories, Medisense Products, Bedford, MA).

#### 2.5.2 Glucagon‐like peptide‐1

Plasma total GLP‐1 (pmol/L) was measured by radioimmunoassay (GLPIT‐36HK, Millipore, Billerica, MA). The minimum detectable limit was 3 pmol/L, and the intra‐assay and inter‐assay coefficients of variation (CVs) were 4.2% and 10.5%, respectively.^26^

#### 2.5.3 Glucose‐dependent insulinotropic polypeptide

Plasma total GIP (pmol/L) was measured by radioimmunoassay with modifications of a published method.^27^ The minimum detectable limit was 2 pmol/L, and the intra‐assay and inter‐assay CVs were 6.1% and 15.4%, respectively.^26^

### 2.6 Statistical analysis

The area under the curve (AUC) was calculated for all variables using the trapezoidal rule. Basal values and AUCs were compared using one‐factor repeated‐measures analysis of variance (ANOVA). Variables were also analysed using two‐factor repeated measures ANOVA, with treatment and time as factors. Where significance was revealed by ANOVAs, post hoc comparisons adjusted by Bonferroni–Holm's correction, were performed. All analyses were performed using SPSS 17.0.0 (SPSS Inc, Chicago, IL). Data are presented as mean values ± SEM. A *P* value of less than .05 was considered significant.

## 3 RESULTS

The studies were well tolerated and completed by all participants. There were no differences in baseline concentrations of blood glucose (*P* = .78), plasma GIP (*P* = .59) or plasma GLP‐1 (*P* = .98) between the four study days.

### 3.1 Blood glucose

**FIGURE 1:**
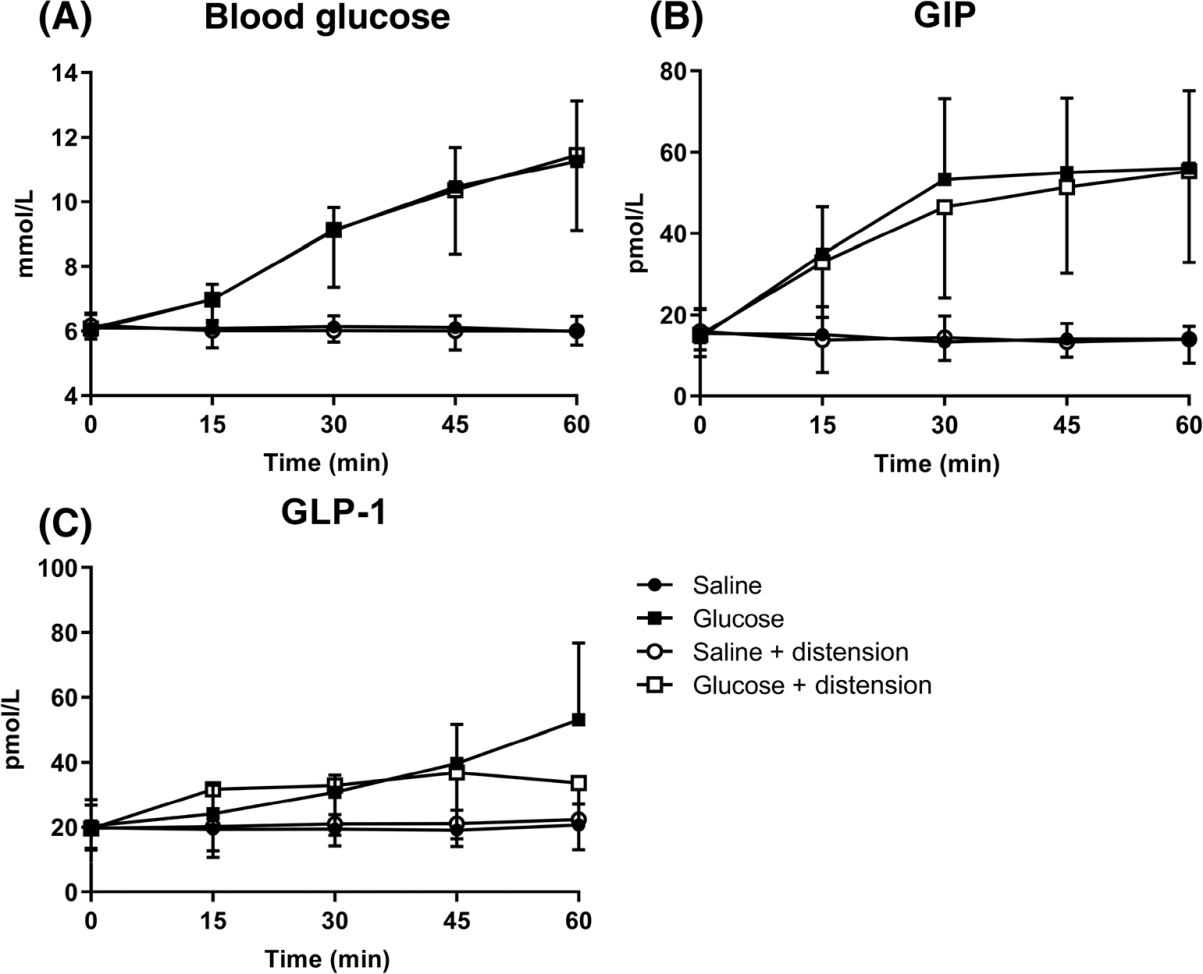
Effects of intraduodenal saline (●, ○) and glucose at 3 kcal/min (■, □) with (○, □) and without (●, ■) gastric distension achieved by a barostat set at 8 mmHg above minimum distending pressure on A, Blood glucose, B, Plasma glucose‐dependent insulinotropic polypeptide (GIP), and C, Glucagon‐like peptide‐1 (GLP‐1) in eight healthy participants. Data are mean ± standard error

There was a predictable increase in blood glucose during glucose and glucose + distension (*P* < .001 for both), and no change during either saline or saline + distension (*P* = .55 and *P* = .48, respectively). There was a treatment effect (*P* < .001) for the AUC for blood glucose, so that blood glucose was greater during glucose compared with saline (*P* < .001) and saline + distension (*P* < .001), and greater during glucose + distension compared with saline (*P* < .005) and saline + distension (*P* < .005), with no difference between glucose and glucose + distension (*P* = 1.00) or between saline and saline + distension (*P* = 1.00) (Figure 1A).

### 3.2 Glucose‐dependent insulinotropic polypeptide

There was a prompt and sustained increase in GIP during glucose and glucose + distension (*P* < .001 for both), and no change during saline and saline + distension (*P* = .59 and *P* = .18, respectively). There was a treatment effect (*P* < .001) for the AUC for GIP, so that GIP was greater during glucose compared with saline (*P* < .001) and saline + distension (*P* < .001), and greater during glucose + distension compared with saline (*P* < .005) and saline + distension (*P* < .005), with no difference between glucose and glucose + distension (*P* = .99) or between saline and saline + distension (*P* = 1.00) (Figure 1B).

### 3.3 Glucagon‐like peptide‐1

There was a gradual and sustained increase in GLP‐1 during glucose and glucose + distension (*P* < .001 and *P* < .05, respectively), and no change during saline and saline with distension (*P* = .67 and *P* = .25, respectively). There was a treatment effect (*P* < .001) for the AUC for GLP‐1, so that GLP‐1 was greater during glucose compared with saline (*P* < .001) and saline + distension (*P* < .005), but not glucose + distension (*P* = 1.00). There was no difference between saline and saline + distension (P = 1.00) or between glucose + distension and saline + distension (*P* = .12) (Figure 1C and Table 1).

**TABLE 1:** Total area under the curve (tAUC) from 0 to 60 minutes for glucose (mmol/L x min), glucose‐dependent insulinotropic polypeptide (GIP) (pmol/L x min) and glucagon‐like peptide‐1 (GLP‐1) (pmol/L x min) following intraduodenal (ID) saline, glucose, saline with balloon distension and glucose with balloon distension (n = 8). Data are mean values ± SEM

	ID saline	ID glucose	ID saline and distension	ID glucose and distension
Glucose tAUC (mmol/L x min)	366 ± 7	530 ± 17	362 ± 9	528 ± 29
GIP tAUC (pmol/L x min)	870 ± 110	2680 ± 330	850 ± 120	2490 ± 360
GLP‐1 tAUC (pmol/L x min)	1170 ± 120	1970 ± 170	1250 ± 160	1920 ± 300

### 3.4 Intrabag volumes

At baseline, the intrabag volume was 443 ± 80 ml during glucose + distension and 410 ± 80 ml for saline + distension and, at 60 minutes, the intrabag volume was 790 ± 71 ml for glucose + distension and 637 ± 80 ml for saline + distension.

## 4 DISCUSSION

Our study indicates that, unlike rodents,^7^, ^8^ gastric distension, with or without small intestinal glucose exposure, does not appear to be a stimulus for either GIP or GLP‐1 secretion in humans. The study was conducted under carefully controlled experimental settings to determine whether there was an effect of gastric distension on GIP or GLP‐1 secretion. Intraduodenal infusion of glucose or saline was used to avoid the confounding effect of variable gastric emptying,^28^ and glucose was administered at a rate that mimics that of gastric emptying^29^ and is known to elicit substantial GLP‐1 and GIP responses.^25^ It is, accordingly, unlikely that our observations would have been modified with a higher rate of nutrient delivery. Our study used the barostat technique, a method accepted as the ‘gold standard’ for controlled gastric distension,^30^ and yielded distension volumes of ~ 700 ml at the given pressure, which was associated with a sensation of fullness.^31^ For comparison, intragastric balloons with volumes of 550 ml are effective for inducing weight loss,^32^ and for adjustable intragastric balloons, volumes are initiated at 400‐550 ml and do not exceed 850 ml.^3^ It should be appreciated that the air‐filled barostat balloon distends predominantly the proximal, rather than the distal, stomach. Distension of the antrum may be of particular importance in satiation^33^ and intragastric balloons positioned in the antrum have been associated with greater weight loss.^34^ However, traditionally, intragastric balloons have been inserted proximally in the fundus,^35^, ^36^ although they may migrate to the antrum.^34^

The reason(s) for the apparent discrepancy in the effect of gastric distension on GLP‐1 secretion between rodents and humans is uncertain. In response to nutrients, the effect of GLP‐1 to increase insulin secretion is evident in both rodents^37^ and humans.^38^ Similarly, GIP has been considered to have a major insulinotropic effect in rodents,^39^ a concept recently consolidated by studies using antagonists to GIP (GIP_3−30_NH_2_) in humans.^40^ A notable difference between rodent and human incretin physiology is that nutrient‐stimulated GLP‐1 secretion is reduced in rodents following subdiaphragmatic vagotomy, while direct vagal stimulation increases GLP‐1 secretion,^41^ indicating that GLP‐1 secretion in rodents is modulated by the vagus nerve. By contrast, humans who have had a truncal vagotomy with pyloroplasty display an increase, rather than a decrease, in nutrient‐stimulated GLP‐1 and GIP secretion, although more rapid gastric emptying as a consequence of the pyloroplasty may well represent a confounder.^42^

Future studies investigating the effect of gastric distension on incretin hormone secretion in individuals with obesity and/or type 2 diabetes would be of interest.

### 4.1 Limitations

Our proof‐of‐concept pilot study had a small sample size, in part because of its technical complexity, and may be underpowered to identify small differences in the effect of gastric distension on GLP‐1 or GIP secretion. Our healthy participants were older (i.e. aged > 65 years), but any reductions in incretin hormone secretion with age are modest, and the stimulation of both GLP‐1 and GIP by intraduodenal glucose remained substantial.^43^ We also cannot exclude the possibility that exposure of the stomach to nutrients (i.e. nutritive distension) in the course of normal meal ingestion may influence incretin secretion.^9^

In conclusion, in healthy, older adults, gastric distension did not stimulate GIP or GLP‐1 secretion either in the absence or presence of small intestinal nutrient exposure.

## AUTHOR CONTRIBUTIONS

RJJ and LGT were involved in the literature review, data processing, data analysis and interpretation. TW was involved in the study design, data analysis and interpretation. SS was involved in the study methodology and data processing. CF‐B, CKR and MH were involved in data analysis and interpretation. KLJ was involved in the study design, conduct, literature review, data analysis and interpretation. All the authors contributed to critical review of the manuscript.

## CONFLICT OF INTEREST

The authors report no conflicts of interest. TW has been supported by a Mid‐Career Fellowship from The Hospital Research Foundation.

### PEER REVIEW

The peer review history for this article is available at https://www.webofscience.com/api/gateway/wos/peer-review/10.1111/dom.15042.

## Data Availability

The data that support the findings of this study are available from the corresponding author upon reasonable request.
